# Supplementary material for: A systematic review of conference papers presented at two large Japanese psychology conferences in 2013 and 2018: did Japanese social psychologists selectively report p < 0.05 results without peer review?
Source: PeerJ. 2024 Jan 18;12:e16763. doi: 10.7717/peerj.16763 (PMC10800096; doi:10.7717/peerj.16763)
Supplement: Supplemental Information 1 — This is an anonymous copy of the systematic review protocol for stage 2, which was preregistered on January 27, 2021, and updated on August 8, 2021. [file peerj-12-16763-s001.pdf]

Supplementary 1

This is an anonymous copy of the systematic review protocol for stage 1 (revision 1), which was preregistered on September 15th, 2020.

ADMINISTRATIVE INFORMATION

Title

A systematic review protocol for meta-analyses of conference papers presented at the Japanese Psychological Association and the Japanese Society for Social Psychology in 2013 and 2018 (stage 1)

Registration

This protocol will be uploaded on our OSF project page: XXXX (Because the OSF project page has links to signed contents, the URL is removed to keep the anonymity of this document)

Authors

Author information has been removed to anonymize the protocol.

Amendment

Upon discussing some unpredicted irregularities among the target papers, we made some minor modifications to the protocol since our first official version of the systematic review protocol. The modifications are made on the following coding variables; “group mean”, “group var plot”, “significant?”, “stats method.” See the “Data Items” section for details. These changes were made in Sep. 11, 2020. Besides, we decided to make this version (stage 1, revision 1) open to the public by sharing the Google Drive URL along with uploading the PDF on the OSF project page, which is still not open to the public. We also make several minor corrections in English writing (grammar, spelling) mainly following the suggestions by Grammarly (grammarly.com).

Support

This study is supported by JSPS KAKENHI Grant Number XXXXX (Grant number was removed to anonymize the protocol). JSPS (Japanese Society for Promotion of Science) provides only financial support and does not play any other roles in developing the protocol.

Conflicts of Interest

There are no conflicts of interest to report.

INTRODUCTION

Background

It has been almost 10 years since psychologists discovered (or re-discovered) the replicability crisis in their discipline. During the years, the (surprisingly or not-surprisingly) low replicability rates of psychological studies published in prestigious journals (i.e., Journal of Personality and Social Psychology, Psychological Science, etc) have been documented (Open Science Collaboration, 2012, 2015) and questionable research practices (QRPs) and p-hacking behind the crisis have been pointed out (Ikeda & Hiraishi, 2016; Simmons et al., 2011). It is argued that the scientifically inappropriate conducts by psychologists are products of publication biases in favor of novel and statistically significant (i.e., $p < .05$) results. The logic is that since psychologists need more publications in more prestigious journals to get promoted, and since journals put a priority on publishing novel, surprising, and significant studies, psychologists resort to QRPs to squeeze publishable $p < .05$ results out of their data.

But, are the journal editors and reviewers solely responsible for the crisis? Given the fact that they are also members of the academic community, the answer cannot be yes. It is much more probable that the psychological community has (or used to have) a culture that values novel, surprising, and significant results and that editors and reviewers just followed the cultural norm when they evaluate the submitted manuscripts. Of course, there is no denying that such actions by the editors and reviewers worsened the scientific integrity of the discipline. Still, we suspect that it is not only journal editors and reviewers who valued novelty and surprisingness too much but also psychologists in general do share the view.

Peculiarities of conference paper format of Japanese psychological societies.

We propose that annual conferences of two Japanese academic societies, the Japanese Psychological Association (JPA) and the Japanese Society for Social Psychology (JSSP), provide interesting and unique resources that can be used to test the hypothesis; Psychologists in general put priority in novelty and statistical significance regardless of the preferences of reviewers. With more than one thousand members (about 8000 for JPA and 1700 for JSSP) the two societies are among the largest academic communities of psychology researchers in Japan. The main presentation format at the annual conference for the two societies is poster presentations. Each year hundreds of posters are presented at the conference; the posters at these conferences are one of the main research outlets for Japanese social psychologists. For

instance, there were 360 poster presentations at the JSSP conference in 2017 whereas only 18 articles were published in the official journal of the society.

There are two peculiar characteristics in the poster formats. First, the authors of a poster must submit a two-column, one-page conference paper in A4 format. Therefore, authors can (and must) report details of the methods and results of their study. While the posters are presented only at the conference venue, the conference papers are archived and made publicly available online by the society. Second, the conference papers do not undergo, in effect, any review process. Therefore, authors do not have to care about the evaluation by the reviewers. Combined, the conference papers of the two societies constitute an archive of what Japanese psychologists have done when they can publicize their study without reviews. We plan to conduct a systematic review of the accumulated records of psychologists' behaviors.

Purpose of the systematic review project

The purpose of the current systematic review project is two folds. The first is to examine the existence and the degree of publication biases in the conference papers for JPA and JSSP. As mentioned above, these two societies do not require peer reviews for the conference presentations. This enables us to test if social psychologists resort to QRPs and *p*-hackings even when they have chances to report their study without any interference from the reviewers. On the other hand, however, the fact that there is no review process for publication means that the quality of writings is not guaranteed. Therefore, it is necessary to ascertain how elaborate the information in the conference papers is. This is what *this* protocol intends to do. We will check and code whether each paper reported details of methods and results, such as sample sizes, *p*-values, means, SDs, statistics, effect sizes, and confidence intervals. After we have finished data collection with this protocol, we will collect the exact values of N, p, and other statistics and conduct *p*-curve analyses and funnel plot analyses to see if publication biases are evident even among the conference papers.

The second purpose of the project is to find out subfields of social psychology where enough information has been accumulated so that we can synthesize the existing data to get aggregate effect sizes. That way, we can present (hopefully less biased) effect size estimates of certain social psychological phenomena in an Eastern society (Japan).

Target studies

We will review the conference papers presented either at the JPA or at the JSSP in 2013 and 2018. Because the numbers of papers presented at the two conferences are enormous, we decided to limit our scope by restricting the target year of presentation; a relatively distant year (2013), where the knowledge about the replicability crisis was not widely shared among Japanese social psychologists, and a more recent year (2018). During the 5 years between 2018 and 2013, several attempts had been made to advertise the crisis to Japanese psychologists; publication of special issues on the replicability crisis in a journal "Japanese Psychological Review" and holding symposia at several academic conferences (including JPA

and JSSP). Therefore, we conjecture that some changes could have occurred during the years in the way social psychologists report their studies.

In the process of review protocol development, it was revealed that there is considerable heterogeneity in the quantity and quality of information written in each conference paper. For instance, statistics reported in papers vary greatly according to whether the study is a questionnaire survey or an experiment. Hence, it is unrealistic to construct a one-fits-all review protocol. Therefore, we decided to focus only on experimental studies, most of which share the ANOVA design and are easy to be covered by one protocol.

Planned analyses

As this protocol describes the very first step of our systematic review project, currently we plan only simple analyses. We will examine the frequencies and detailedness at which several statistical information (e.g., p-values, sample sizes, means, etc) are reported in the conference papers. In the following steps, we will conduct p-curve analyses, funnel plot analyses, and more.

METHODS

Eligibility criteria

We first identify all conference papers accompanying poster presentations at the Japanese Society for Social Psychology (JSSP) annual conferences in 2013 and 2018. As for the Japanese Psychological Association (JPA), conference papers accompanying the posters in the “Society and/or Culture” field for the 2013 and 2018 conferences are identified. We will select the papers with two eligibility criteria. First, whether the study was experimental or not. Second, whether the study presented directed predictions in the introduction section (see “selection process” section for detail).

Information sources

Full text of papers for the JPA conference are available online at https://www.jstage.jst.go.jp/browse/pacjpa/-char/ja (since 2006).

Full text of papers for JSSP conferences are available online at http://iap-jp.org/jssp/conf_archive/ (since 2010).

Search strategy

We will download all the relevant papers from the JPA and JSSP archives of conference papers.

Study Records

Data management

All PDF files of the conference papers are downloaded via the Internet and stored in a private server managed by one of the authors (AM) and each PDF file is given a unique URL. We created a coding sheet (google spreadsheet file) on which the URLs of target papers are listed. The coding sheet is located under a Google Drive owned by the guarantor (KH) under the XXXX (anonymized) domain (G suite for education), which is provided by KH's affiliation (XXXX; anonymized). The file is shared among the project members. The version history of the coding sheet is automatically saved by the G Suite for Education service.

Two coders (MS and DN) record the codings on the sheet. As we plan to do coding at the effect level, each row of the coding sheet corresponds to each effect. Thus, a paper/study with multiple effects occupies multiple rows in the sheet.

Shared folder on Google Drive

XXXX (anonymized)

Coding sheet (Google spreadsheet)

XXXX

Selection process

The study identification process is rather mechanical because all of the potential target papers are archived by the academic society (JPA and JSSP). As we have written above, all of the conference papers in 2013 and 2018 are identified for JSSP. As for JPA, all the papers in the "Society and/or Culture" field for 2013 and 2018 conferences are identified. One of the members (AM) and her assistant identified the papers and downloaded the PDF files onto our storage space.

Collected studies are assessed by two independent coders (MS and DN) for their eligibility. There are two stages of selection. The first selection will be done at the study level. That is, each study in a paper will be assessed for its eligibility. Therefore, study 1 of a paper may be excluded while study 2 of the same paper is included in the review. The coders read the paper and determine whether each study is experimental or not. The second stage of selection will be done at the effect level. The coders determine whether each effect reported in the introduction has a directed prediction or not (see below for details).

By saying "experimental", we mean that at least one factor was controlled by the researchers. As such, a study that has two and only two naturally occurring factors (e.g., culture and gender) will be excluded. In the same way, a study that compared a high-trait-anxiety group and a low-trait-anxiety group is not treated as experimental no matter how complex techniques or

technologies (e.g., IAT, fMRI) are employed. On the other hand, we include vignette studies as far as one or more factors are controlled by the researcher.

For an experimental study, we will code whether it has a directed prediction(s) for the effects that correspond to its main research questions. For instance, in a 2 (X1a vs. X1b) x 2 (X2a vs. X2b) design, there are three effects (two main effects and one interaction), each of which can be a target of the research question. If the paper explicitly states a prediction for the main effect of X1 (e.g., X1a > X1b), it will be coded as having a directed prediction. If the paper just proposed that there could be a difference between X2a and X2b but no clear statement about the direction of the effect, it will be coded as non-directed. For the interaction term, if the paper clearly stated either the attenuation (e.g., X2 attenuates the effect of X1 on Y) or the reversal of the effects (X1a > X1b for X2a whereas X1a < X1b for X2b), it will be coded as directed. Otherwise, it will be coded as non-directed. If there is no prediction (e.g., predictions are made only for two main effects but no mention is given for the interaction), it will be coded as no prediction. When an independent variable X has more than 2 levels and the authors provide explicit predictions regarding the pairwise comparisons (Xa > Xc and Xb > Xc), we code all the pairwise comparisons as having directed predictions. If there was no mention of the main effect of X, we do not code it; we will code only the pairwise comparisons.

If a study is a replication of a previously reported effect (e.g., Xa > Xb), the effect is coded as having a directed prediction regardless of whether it was a direct replication or a conceptual one. However, if a study exploratory examined the boundary condition of a previously reported effect (i.e., to see if Xa > Xb holds in condition A, B, C,...or, K) without any theoretical predictions, that will be coded as an interaction with non-directed prediction. If a study examined two (or more) competing predictions (Xa > Xb or Xa < Xb), as far as those predictions were theoretically grounded, it will be coded as having directed predictions.

The coders determine the “directedness” of predictions based solely on the descriptions in the introduction section of the paper. When the writing is ambiguous (i.e., “we suspect that there is a possibility that it may be the case that X1 > X2 could hold...”), we will mark it on the coding sheet and discuss later among the project members.

In a sense, the second selection stage can be regarded as “coding” as we plan to examine the frequency of effects with and without directed predictions in the analyses step. On the other hand, this process is a selection because we plan to collect detailed information only from the effects with directed predictions.

Data collection process

Data collection protocol has been developed through several pilot collections. The first three pilot collections and coding were conducted by the initial members of the project (KH, AM, MH, and YF) and they designed the alpha version of the protocol. Two coders (MS and DN) conducted additional two pilot selections and the beta protocol was proposed. The official version was finalized by conducting selection and coding on randomly selected 100 papers from the target papers in 2018 (499 papers from JPA and JSSP in total). Any discrepancies between

the two coders at this stage were discussed and resolved and reflected in the final version of the protocol. Two coders split the remaining 399 papers in half and conduct the selection and coding. We do not plan to obtain any confirming data from the authors of the conference papers, at least at the current stage of the project. This is because we are interested in the psychologists' styles in reporting the studies in conference papers.

The two coders finished coding the 499 papers by September 11, 2020. On the day, the project members discussed some unclear aspects of the coding and decided to make some amendments to the protocol (revision 1). The details of the discussion can be found on the project progress record (in Japanese; the URL link to the Google document file is removed to anonymize the protocol).

Data items

We will code the below variables from the target papers.

Research ID

ID code for each paper. The IDs are given preceding the coding.

rated by

Reviewers ID (XXXX or YYYY) *Reviewers' names are removed to anonymize the protocol.

Study ID

When a paper has more than one study, each will be given an id number.

Experiment?

Whether the study was an experiment or not. Only when the study is experimental, the following variables will be coded. (Yes/No)

Hypothesis testing?

Whether the effect in question has a directed prediction or not. Only when this variable is coded “directed”, the following variables will be coded. (directed, non-directed, nothing)

Effect ID

If the experiment had more than one effect to be reported, each will be given an ID number.

Effect type

Specify the type of the effect. Will be selected from the following list (main effect, interaction, simple main effect, mediation, planned comparison, other)

Stat. method

Specify the statistical method utilized to test the hypothesis. Will be selected from the following list. (t, anova, regression, glm, correlation, Bayes factor, SEM, path, mediation, other)

When no information is given as to what kind of analysis was employed to test the declared hypothesis, it will be coded as “absent” (added on 09/11/2020).

Covariate

Whether any covariates are controlled in the analysis. If the interaction with the covariate is described in the introduction, the effect will be given an independent row on the coding sheet. (Present/Absent/Other)

N

Specify whether or not the total sample size is described in the text. (Present/Absent/Other)

n

Specify whether the sample sizes of subgroups are described in the text (Present/Absent/Other).

Stats

Specify whether the statistic (*t*, *F*, Chi-sq, etc) is described in the text (Present/Absent/Other).

Df

Specify whether the degree of freedoms of the statistics are described in the text (Present/Absent/Other)

p

Specify whether the p-value is described in the text. If no information is given, code as “absent”. If only the alpha level is given (e.g., $p < .05$), code as “alpha level”. If the exact p-value is given, code as “present”. Otherwise, code as “other”.

***p*-value**

If the exact p-value is given in the text, record the stats here.

Significant

Specify whether the effect was significant or not (Absent, Significant, Marginally Significant, Not Significant, or other). Only when the author of the paper described the effect as “marginally significant” code as so.

In the case where the hypothesis testing was done via model selections and the relevant path was removed from the final model, this item will be coded as “other” rather than “Significant” (added on 09/11/2020).

effect size value

Specify whether effect sizes are reported in the text or not (Present/Absent/Other).

effect size type

If present on the above variable, write down the type of effect size reported (Cohen d (Hedge's g), r, beta(reg), B(reg), rho, ita^2 (partial ita^2), R^2 (adj. R^2), Cohen f, other).

effect size var info.

Specify whether the variance of the effect sizes are reported in the text or not (Present/Absent/Other).

group mean info.

Specify whether the group means are reported in the text or not (Present/Absent/Other).

When rates or frequencies of particular responses rather than means are presented in the paper, code them as “other” (added on 09/11/2020).

group var info.

Specify whether the group variances are reported in the text or not (Present/Absent/Other).

group mean plot

Specify whether the group means are graphically reported or not (Present/Absent/Other).

group var plot

Specify whether the group variances are graphically reported or not (Present/Absent/Other).

When error-bars are plotted but the kind is not specified (i.e., SD, SE, or CI), code them as “Unknown” (added on 09/11/2020).

Memo

Any additional information worth mentioning is reported here.

Outcomes and prioritization

Not applicable.

Risk of bias in individual studies

Not applicable.

DATA Synthesis

For the current protocols, we will look only at simple frequencies of each variable, and no synthetic analyses will be conducted.

Meta-bias(es)

Because this protocol is preparation for analyzing meta-biases in the next stage of the project, not applicable to the current protocol. We will look at *p*-curves (Simonsohn et al., 2014) and funnel plots in the next stage.

Confidence in cumulative evidence

Because this protocol is preparation for analyzing meta-biases in the next stage of the project, this is not applicable to the current protocol. In the following *p*-curve analyses and funnel plot analyses, the certainty of the results will be judged using the same classification as the GRADE (Welch et al., 2017) approach (i.e., very low, low, moderate, high). It should be noted that there is no system equivalent to GRADE in psychology that can be used to transparently rate and judge the certainty of the results. Therefore we will only utilize the classification and general ideas of GRADE.

Answer the following final questions:

Has data collection begun for this project?

- ~~**No, data collection has not begun**~~
- **Yes, data collection is underway or complete.**
  Specifically, the official protocol finalization started on 03/13/2020 and finished on 06/13/2020. The coding started on the same day and plans to finish by 09/11/2020.

If data collection has begun, have you looked at the data?

- ~~**Yes**~~
- **No**

The (estimated) start and end dates for this project are (optional):

Start date: 2020/03/13

End date: 2020/09/11

Any additional comments before I pre-register this project (optional):

No additional comments.

REFERENCES

Ikeda, K., & Hiraishi, K. (2016). The reproducibility crisis in psychology: Its structure and solutions. *Japanese Psychological Review*, *59*(1), 1–12.

Open Science Collaboration. (2012). An Open, Large-Scale, Collaborative Effort to Estimate the Reproducibility of Psychological Science. *Perspectives on Psychological Science: A Journal of the Association for Psychological Science*, *7*(6), 657–660. https://doi.org/10.1177/1745691612462588

Open Science Collaboration. (2015). Estimating the reproducibility of psychological science. *Science*, *349*(6251), aac4716–1 – aac4716–aac4718. https://doi.org/10.1126/science.aac4716

Simmons, J. P., Nelson, L. D., & Simonsohn, U. (2011). False-positive psychology: undisclosed flexibility in data collection and analysis allows presenting anything as significant. *Psychological Science*, *22*(11), 1359–1366. https://doi.org/10.1177/0956797611417632

Simonsohn, U., Nelson, L. D., & Simmons, J. P. (2014). P-curve: A key to the file-drawer. *Journal of Experimental Psychology. General*, *143*(2), 534–547. https://doi.org/10.1037/a0033242

Welch, V. A., Akl, E. A., Guyatt, G., Pottie, K., Eslava-Schmalbach, J., Ansari, M. T., de Beer, H., Briel, M., Dans, T., Dans, I., Hultcrantz, M., Jull, J., Katikireddi, S. V., Meerpohl, J., Morton, R., Mosdol, A., Petkovic, J., Schünemann, H. J., Sharaf, R. N., … Tugwell, P. (2017). GRADE equity guidelines 1: considering health equity in GRADE guideline development: introduction and rationale. *Journal of Clinical Epidemiology*, *90*, 59–67. https://doi.org/10.1016/j.jclinepi.2017.01.014
