# Supplementary material for: A systematic review of conference papers presented at two large Japanese psychology conferences in 2013 and 2018: did Japanese social psychologists selectively report p < 0.05 results without peer review?
Source: PeerJ. 2024 Jan 18;12:e16763. doi: 10.7717/peerj.16763 (PMC10800096; doi:10.7717/peerj.16763)
Supplement: Supplemental Information 2 — This is an anonymous copy of the systematic review protocol for stage 2, which was preregistered on January 27, 2021, and updated on August 8, 2021. [file peerj-12-16763-s002.pdf]

Supplementary 2

This is an anonymous copy of the systematic review protocol for stage 2, which was preregistered on January 27, 2021, and updated on August 8, 2021.

ADMINISTRATIVE INFORMATION

Title

A systematic review protocol for meta-analyses of conference papers presented at the Japanese Psychological Association and the Japanese Society for Social Psychology in 2013 and 2018 (stage 2)

Registration

This protocol will be uploaded on our OSF project page: XXXX (Because the OSF project page has links to signed contents, the URL is removed to keep the anonymity of this document)

Authors

Author information has been removed to anonymize the protocol.

Amendment

We updated our analysis and interpretation plan of z-curve analysis on August 8, 2021 (section METHODS - DATA synthesis - Z-curve analysis). As of that date. we have finished data collection but have not analyzed the data.

Support

This study is supported by JSPS KAKENHI Grant Number XXXX (Grant number was removed to anonymize the protocol) awarded to KH, AM, MH, and YF. JSPS (Japanese Society for Promotion of Science) provides only financial support and does not play any other roles in developing the protocol.

Conflicts of Interest

There are no conflicts of interest to report.

INTRODUCTION

Background

It has been almost 10 years since psychologists discovered (or re-discovered) the replicability crisis in their discipline. During the years, the (surprisingly or not-surprisingly) low replicability rates of psychological studies published in prestigious journals (i.e., Journal of Personality and Social Psychology, Psychological Science, etc) have been documented (Open Science Collaboration, 2012, 2015) and questionable research practices (QRPs) and p-hacking behind the crisis have been pointed out (Ikeda & Hiraishi, 2016; Simmons et al., 2011). It is argued that the scientifically inappropriate conducts by psychologists are products of publication biases in favor of novel and statistically significant (i.e., $p < .05$) results. The logic is that since psychologists need more publications in more prestigious journals to get promoted, and since journals put a priority on publishing novel, surprising, and statistically significant studies, psychologists resort to QRPs to squeeze publishable $p < .05$ results out of their data.

But, are the journal editors and reviewers solely responsible for the crisis? Given the fact that they are also members of the academic community, the answer can hardly be yes. It is much more probable that the psychological community has (or used to have) a culture that values novel, surprising, and statistically significant results and that editors and reviewers just followed the cultural norm when they evaluate the submitted manuscripts. Of course, there is no denying that such actions by the editors and reviewers worsened the scientific integrity of the discipline. Still, we suspect that it is not only journal editors and reviewers who valued novelty and surprisingness too much but also psychologists in general do share the view. The current study aims to test the hypothesis that psychologists in general have (or used to have) the tendency to pursue novel, surprising, and statistically significant results and have been resorting to QPRs irrespective of the existence of reviewers and editors forcing them to report such results.

Peculiarities of conference paper format of Japanese psychological societies.

We propose that annual conferences of two Japanese academic societies, the Japanese Psychological Association (JPA) and the Japanese Society for Social Psychology (JSSP), provide interesting and unique resources to test the hypothesis; Psychologists in general put priority on novelty and statistical significance regardless of the preferences of reviewers. With more than one thousand members (about 8,000 for JPA and 1,700 for JSSP) the two societies are among the largest academic communities of psychology researchers in Japan. The main presentation format at the annual conference for the two societies is poster presentations. Each year hundreds of posters are presented at the conference; the posters at these conferences are one of the main research outlets for Japanese social psychologists. For instance, there were 360 poster presentations at the JSSP conference in 2017 whereas only 18 articles were published in the official journal of the society.

There are two peculiar characteristics in the poster formats. First, the authors of a poster must submit a two-column, one-page conference paper in A4 format. Therefore, authors can (and must) report details of the hypotheses, methods, and results of their study. While the posters are presented only at the conference venue, the conference papers are archived and made publicly available online by the society. Those conference papers are often included in the C.V.s and are used in the evaluation of researchers' annual achievements. Second, the conference papers do not undergo, in effect, any review process. Therefore, authors do not have to care about the evaluation by the reviewers. Combined, the conference papers of the two societies constitute an archive of what Japanese psychologists have been doing when they can publicize their studies without reviews. We plan to conduct a systematic review of the accumulated records of psychologists' behaviors.

Purpose of the systematic review project

The purpose of the current systematic review project is two folds. The first is to examine the existence and the degree of publication biases in the conference papers for JPA and JSSP. As mentioned above, these two societies do not require peer reviews for the conference presentations. This enables us to test if social psychologists in Japan ever resorted to QRPs even when they could report their studies without any interference from the reviewers. On the other hand, however, the fact that there is no review process for publication means that the quality of writings is not guaranteed. Therefore, it is necessary to ascertain how elaborate the information in the conference papers is. This is what the first stage of the systematic review protocol intended to do (https://osf.io/m7evs). We check and code whether each paper reported details of hypotheses, predictions, methods, and results, such as sample sizes, *p*-values, means, SDs, statistics, effect sizes, and confidence intervals.

After we finish the initial data collection with the stage 1 protocol, we proceed to the stage 2 protocol (this document) where we collect the exact statistical values such as *p*-values, *t*-values, *F*-values, DFs, etc. This enables us to conduct *p*-curve and z-curve analyses (Bartoš & Schimmack, 2020; Brunner & Schimmack, 2020; Simonsohn et al., 2014a, 2014b, 2015; van Aert et al., 2016). The *p*/*z*-curve analyses are the type of meta-analysis that rely solely on the reported *p*-values. With the *p*/*z*-curve analyses, we can estimate evidential values of a set of target studies. It should be noted that *p*/*z*-analyses do not directly reveal the existence of QRPs such as selective reporting, *p*-hacking, etc (especially so with the z-curve analyses (Brunner & Schimmack, 2020). We believe, though, that we can indirectly examine the appropriateness of the researcher's behavior via the estimation of the evidential value of the conference papers.

In the current study, we plan to estimate only evidential value even though estimation of aggregated effect size is possible with the *p*/*z*-curve analyses. This is because, due to the rather wide variations in topics and fields of the target papers, it is almost meaningless to estimate the "aggregated" effect size from them.

The second purpose of the project is to find out subfields of social psychology where enough information has been accumulated so that we can synthesize the existing data to get aggregate

effect sizes. That way, we can present (hopefully less biased) effect size estimates of certain social psychological phenomena in an Eastern society (Japan). This will be achieved with another systematic review protocol.

Target studies

We review the conference papers presented either at the JPA or at the JSSP in 2013 and 2018. Because the numbers of papers presented at the two conferences are enormous, we decided to limit our scope by restricting the target year of presentation; a relatively distant year (2013), where the knowledge about the replicability crisis was not widely shared among Japanese social psychologists, and a more recent year (2018). During the 5 years between 2018 and 2013, several attempts had been made to advertise the crisis to Japanese psychologists; publication of special issues on the replicability crisis in the journal *Japanese Psychological Review* (Miura et al., 2018, 2019; Tomonaga et al., 2016) and holding symposia at several academic conferences (including JPA and JSSP). Therefore, we conjecture that some changes could have occurred during the years in the way social psychologists report their studies.

In the process of review protocol development, it was revealed that there is considerable heterogeneity in the quantity and quality of information written in each conference paper. For instance, statistics reported in papers vary greatly according to whether the study is a questionnaire survey or an experiment. Hence, it is unrealistic to construct a one-fits-all review protocol. Therefore, we decided to focus only on experimental studies, most of which share the ANOVA design and are easy to be covered by one protocol.

Among the conference papers reporting experimental studies, we will include those papers that describe details of statistical values sufficient enough to conduct *p*-curve and *z*-curve analysis. See the methods section for details.

METHODS

Eligibility criteria

We first identify all conference papers accompanying poster presentations at the Japanese Society for Social Psychology (JSSP) annual conferences in 2013 and 2018. As for the Japanese Psychological Association (JPA), conference papers accompanying the posters in the “Society and/or Culture” field for the 2013 and 2018 conferences are identified. At the first stage of the protocol, we select the papers with two eligibility criteria (https://osf.io/m7evs). First, whether the study was experimental or not. Second, whether the study presented directed predictions in the introduction section (see the selection process section for detail).

In addition to the two criteria, we have an additional criterion in the current stage 2 protocol; whether the paper reports statistical information sufficient to compute p-value (hereafter, *p*-stats) or *p*-values. The *p*-values included in a *p*-curve analysis should be independent of each other.

Therefore, we will take the *p*-value that first appeared in each paper. Besides, the proposers of *p*-curve analysis strongly recommend computing *p*-values from statistical values (e.g., *t*-value and degrees of freedom in case of *t*-test) rather than relying on *p*-values reported by the authors of the target articles (Simonsohn et al., 2015). Thus, the *p*-curve analyses examine only those studies that report sufficient statistical information (*p*-stats, Figure 1). However, due to the limited space available for each conference paper (one page in A4 format), authors sometimes omit the detailed statistical information and report only *p*-values. That means we may not be able to have a sufficient sample size with the strict eligibility criteria. Thus, as for *z*-curve analyses, we will include both studies with *p*-stats and studies only with *p*-values in order to have larger statistical power (Figure 1). A paper without exact *p*-values but only report the alpha level (e.g., $p < .05$) is not eligible for the current meta-analysis.

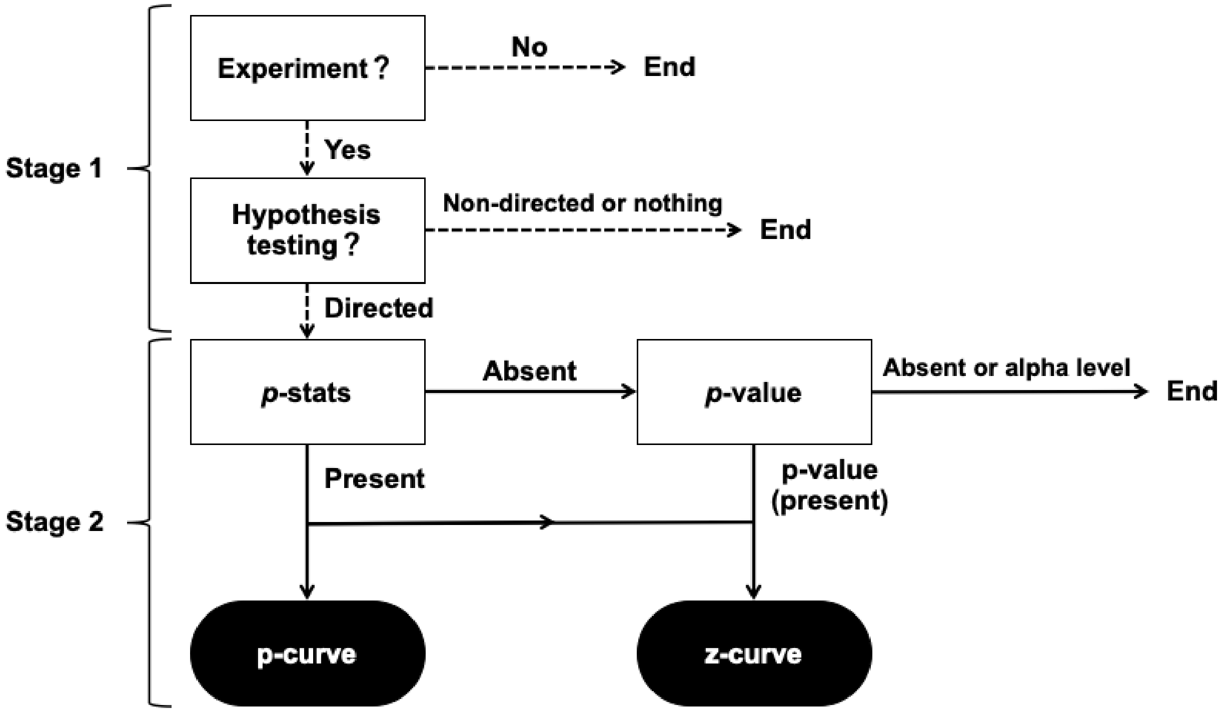

Figure 1. Flowchart of the selection process. Dashed lines indicate selection flows in stage 1 protocol whereas solid lines indicate selection process in stage 2 protocol. The "*p*-stats" denotes detailed statistical information sufficient to compute *p*-values. When *p*-stats are not reported in the paper but exact *p*-value (e.g., $p = .034$) is reported, we will use it in the z-curve analyses. Those conference papers that report only alpha levels (e.g., $p < .05$) will be excluded from the meta-analysis. We will use the *p*-stats/p-values first reported in a paper in the *p*-curve and *z*-curve analyses. For sensitivity analyses, we will use the second *p*-stats/*p*-value in the papers when they are available. We will also use studies only with p-stats to conduct z-curve analyses as another set of sensitivity analyses.

Information sources

Full text of papers for the JPA conference is available online at https://www.jstage.jst.go.jp/browse/pacjpa/-char/en (since 2006).

Full text of papers for JSSP conferences is available online at http://iap-jp.org/jssp/conf_archive/ (since 2010).

Search strategy

We will download all the relevant papers from the JPA and JSSP archives of conference papers.

Study Records

Data management

All PDF files of the conference papers are downloaded via the Internet and stored in a private server managed by one of the authors (AM) and each PDF file is given a unique URL. We created a coding sheet (google spreadsheet file) on which the URLs of target papers are listed. The coding sheet is located under a Google Drive owned by the guarantor (KH) under the XXXX (anonymized) domain (G suite for education), which is provided by KH's affiliation (XXXX; anonymized). The file is shared among the project members. The version history of the coding sheet is automatically saved by the G Suite for Education service.

Two coders (MS and DN) record the codings on the sheet. As we plan to do coding at the effect level, each row of the coding sheet corresponds to each effect. Thus, a paper/study with multiple effects occupies multiple rows in the sheet.

A shared folder on Google Drive

XXXX (anonymized)

Coding sheet (Google spreadsheet)

XXXX (anonymized)

Selection process

The study identification process is rather mechanical because all of the potential target papers are archived by the academic society (JPA and JSSP). As we have written above, all of the conference papers in 2013 and 2018 are identified for JSSP. As for JPA, all the papers in the "Society and/or Culture" field for the 2013 and the 2018 conferences are identified. One of the members (AM) and her assistant identified the papers and downloaded the PDF files onto our storage space.

Collected studies are assessed by two independent coders (MS and DN) for their eligibility. There are three steps of selection. The first and the second steps are done with the 1st stage of the systematic review protocol (Supplementary 1). In brief, we select conference papers that report experimental studies (1st step) and then select studies that tested the directed hypothesis (2nd step). For details, please see the first stage protocol.

The third step is done with the stage 2 protocol. We will collect either detailed statistical information (*p*-stats) or *p*-values or both. When there are multiple studies with multiple effects reported in one conference paper, we will collect the *p*-stats and/or *p*-value that first appeared in the main text of the paper for the primary analyses. We also collect the second and subsequent *p*-stats and *p*-value for the sensitivity analyses. The *p*-stats will be coded in the format specified by p-curve.com (Figure X2).

```
t(88)=2.1
r(147)=.246
F(1,100)=9.1
f(2,210)=4.45
Z=3.45
chi2(1)=9.1
r(77)=.47
chi2(2)=8.74
```

Figure 2. *p*-stats coding format from p-curve.com.

Data collection process

Data collection protocol has been developed through several pilot collections. The first three pilot collections and coding were conducted by the initial members of the project (KH, AM, MH, and YF) and they designed the alpha version of the stage 1 protocol. Two coders (MS and DN) conducted additional two pilot selections and the beta version was proposed. The official version of the stage 1 protocol was finalized by conducting selection and coding on randomly selected 100 papers from the target papers in 2018 (499 papers from JPA and JSSP in total). Any discrepancies between the two coders at this stage were discussed and resolved and reflected in the final version of the protocol. Two coders split the remaining 399 papers in half and conduct the selection and coding. Discrepancies between the two coders were, again, checked and resolved. That resulted in stage 1 revision 1 of the protocol[1]. After that, the coders conduct

[1] The two coders finished coding the 499 papers from year 2018 by September 11, 2020. On the day, the project members discussed some unclear aspects of the coding and decided to make some amendments to the 1st stage protocol (stage 1, revision 1). The details of the discussion can be found on the project progress record (in Japanese; the link to the shared document file is removed to keep anonymity of the current document). Additional minor clarifications on selection and coding processes are made on December 11, 2020. Most importantly, we clarified

the selection and coding of the target conference papers in 2013 (690 papers from JPA and JSPS in total). Two coders randomly split the 690 papers in half and conduct the selection and coding independently.

We do not plan to obtain any confirming data from the authors of the conference papers, at least at the current stage of the project. This is because we are interested in the psychologists' styles in reporting the studies in conference papers.

Data items

In addition to the variables coded by the stage 1 protocol, we will collect the *p*-stats and *p*-value of each effect. See figure X2 for the *p*-stats. See stage 1 protocol for other variables (Supplementary 1).

Outcomes and prioritization

Not applicable.

Risk of bias in individual studies

Not applicable.

DATA Synthesis

As noted above, we plan to conduct *p*-curve analyses (Simonsohn et al., 2014a, 2014b, 2015) and *z*-curve analyses (Bartoš & Schimmack, 2020; Brunner & Schimmack, 2020).

P-curve analysis

With the *p*-curve analysis, we can test if a set of studies as a whole has evidential value. Specifically, if a set of studies has an evidential value, the *p*-curve (distribution of reported *p*-values smaller than .05) should be right skewed. On the other hand, if the effect in question is null, the *p*-curve should be uniform. Even worse, if researchers resort to *p*-hacking to acquire $p < .05$ results, there will be more *p*-values accumulated just under the .05 criterion (e.g., $p = .048$), leading to left skewed *p*-curve. Given the logic, the original *p*-curve paper proposed to test the right skewness of the full *p*-curve (distribution of all *p*-values under .05). However, several weaknesses of the original idea have been pointed out since its publication (Ulrich & Miller, 2015). For instance, the full *p*-curve analysis is vulnerable to “ambitious” *p*-hackers who try to have *p*-values much smaller than .05 (e.g., $p < .03$).

---

what counts as the “first” and “second” in coding *p*-stats and *p*-value. It was agreed that “first” and “second” are defined in terms of the order of appearance in the main text of a paper, not in terms of the importance of the particular test that produced the statistical values.

The “Better *p*-curve” has been proposed (Simonsohn et al., 2015) and implemented on the website (p-curve.com) to tackle the problem by utilizing the half *p*-curve (distribution of *p*-values under .025). Therefore, we will follow the procedures recommended by the Better *p*-curve. First, we will test the right skewness of the full and half *p*-curves. When the half *p*-curve test is right skewed with $p < .05$ or both the full and half p-curves are right skewed with $p < .1$, we will conclude that the set of studies has evidential value.

When the right-skew tests turn out to be non-significant, we will proceed to the 33% power test; to see if the *p*-curve is flatter than one would expect if the studies were powered at 33%. Since the shape of a *p*-curve depends on the power, the *p*-curve of 33% powered studies would be fairly flat, albeit right-skewed. Therefore, if the observed *p*-curve is significantly flatter than the 33% *p*-curve, we can conclude that the set of studies practically lack evidential value. Following the description on the p-curve.com, we will conclude that the set of studies lack evidential value when 33% power test of the full *p*-curve is $p < .05$ or both the half p-curve and binomial 33% power tests are $p < .1$ (the binomial test examine the share of *p*-values smaller than .025 among all *p*-values under .05).

As noted above, the proposers of *p*-curve analysis strongly recommend to re-calculate *p*-values from *p*-stats such as t-values and DFs (p-stats). Therefore, we will use the p-stats to conduct p-curve analyses (see Figure 2 for the examples of *p*-stats).

Since the *p*-curve analysis has been constantly updated, we will employ the latest version available on the website (p-curve.com) at the time of analysis. If there happens to be a major update during the analysis, we may proceed with the older version, though. We will report the *p*-curve version anyway. Along with the results, we will make the collected *p*-values publicly available so that anyone can examine the data with other versions of the p-curve analysis.

We do not think it is meaningful to estimate the aggregated effect size for the current meta-analysis even though it is possible with the *p*-curve analysis (Simonsohn et al., 2014b; van Assen et al., 2015).

Z-curve analysis

With the z-curve analysis, we can estimate the mean power of a set of studies. As the power is a function of the effect sizes and the sample sizes, small power means small effect size or small sample size or both; lack of evidential value at any rate. It should be noted that there are two types of “mean power.” One is the Expected Replication Rate (ERR) that is the expected percentage of significant studies among exact direct replication of the target study. Put differently, it is the percentage of successful replications if ever we could conduct the exact copies of the original studies. The other is the Expected Discovery Rate (EDR), which is the percentage of significant studies among all the studies that have ever been conducted. The original z-curve (z-curve 1.0) paper proposed a method to estimate the ERR based on reported *p*-values (Brunner & Schimmack, 2020). The updated version (z-curve 2.0) can estimate the

EDR as well (Bartoš & Schimmack, 2020). Also, with the z-curve 2.0, we can estimate the bootstrapped confidence intervals of the mean powers.

Both z-curve 1.0 and z-curve 2.0 can be conducted with an R package (zcurve), that we will use in our analysis. Three methods have been proposed to estimate mean power and implemented in the zcurve package (Kernel Density 1, Kernel Density 2, and Expectation Maximization; KD1, KD2, and EM for short). Specifically, these are different methods to fit the observed z-curve to a shifted standard normal distribution (the size of the shift corresponds to the mean power). We will report the results from all three methods. It is noted that KD1 was proposed in the z-curve 1.0 paper while KD2 and EM were proposed in the z-curve 2.0 paper. We will use the latest version of the zcurve package available at the time of analysis. If there is a major update in the package during the analysis, we may proceed with the older version. We will report the version of the package in any case.

As for the z-curve analyses, we will include both studies with *p*-stats and studies only with *p*-values in order to have larger power from the larger sample sizes. We will also conduct secondary z-curve analyses using only studies *p*-stats as sensitivity analyses. We do not include studies that reported only alpha levels in the current meta-analyses.

The following four sentences are added on August 4, 2021

We will follow Schimmack (2020) in our interpretation of Z-curve analyses. We will compute the point estimates of ERR and EDR and their 95% confidence intervals. Then we will compare the ERR and the EDR with the ODR (observed discovery rate) that is a percentage of significant ($p < .05$) studies among all eligible studies included in our meta-analysis.

If the 95% confidence intervals of ERR do not include the ODR, we will conclude that the possibility of successfully replicating the reported effects is significantly less than what is expected from the ODR. ERR is an expected successful replication rate of exact replication of original studies (i.e., exactly the same sample, time, place, etc.), which is practically impossible. It follows that ERR is an optimistic estimation of replication success. Therefore, if ERR is small than ODR, we conclude that we are less likely to replicate psychological studies reported at JPA and JSSP conferences. That means that the replication crisis in psychology is not yet over, at least in Japanese social psychological society.

If the 95% confidence intervals of EDR do not include the ODR, we will conclude that we have strong evidence of publication bias. ODR is an estimation of the proportion of significant studies among all studies that have ever been conducted. If there is no publication bias, EDR should be the same as ODR. If EDR is smaller than ODR, it indicates that the authors selectively reported those studies with significant *p*-values (i.e., $p < .05$) at the conference.

Using the EDR, we can estimate the maximum FDR (false discovery rate) with Sorić (1989)'s formula. FDR is a proportion of false positive studies among the studies that have been reported to be statistically significant. Even though higher FDR (e.g., FDR > .5) does not necessarily mean improper research conducts by the researchers, if it ever had been observed, it suggests that something may be going wrong in the field. Therefore, we will report the FDR that can be computed with the Z-curve analysis.

Family-wise error control.

As for the *p*-curve analysis, we will conduct several null hypothesis significance tests. We will analyze the 2013 data and the 2018 data separately. For each year's dataset, we will conduct several right-skew tests with different alpha levels. Also, when those tests do not reach significance, we will proceed to the 33% power tests, which, again, include several null hypothesis tests with different alpha levels. As such, controlling for the family-wise error rate is rather complicated. Therefore we decided not to declare any family-wise error correction beforehand. Instead, we declare to report all tests and all raw *p*-values from the p-curve analyses. If the findings are not robust, it should eventually appear in the results (e.g., only a small portion of the tests turns out to be significant) (Lakens, 2020).

Sensitivity analysis

Not a small proportion of conference papers report more than two *p*-stats/*p*-values. We will use the second reported *p*-stats/*p*-values in the papers for sensitivity analyses. We will replace the first *p*-stats/*p*-value with the second *p*-stats/*p*-value when the latter is available. In cases where only one eligible *p*-value is reported in a paper, we will keep using it in the sensitivity analyses. Therefore sample size (number of studies included in the meta-analysis) will be the same in the sensitivity analyses.

Another set of sensitivity analyses include z-curve analyses with studies with p-stats. This is to see if we will have the same levels of evidential values when we have stricter eligibility criteria and to have only those studies that reported quite detailed information as targets.

Meta-bias(es)

This protocol examines the very existence of meta-bias in the reported conference papers. That is, this protocol intends to test evidential values of studies reported in two large Japanese psychological societies. If the studies are biased due to publication bias, selective reporting, or *p*-hackings, it will be revealed by the current analyses. However, it does not mean that the current protocol escapes meta-biases in the broader sense. We will look only at studies reported at Japanese academic conferences, in social psychology. and with experimental manipulations. These restrictions are set because of the availability of suitable data and the feasibility of data collection. Nevertheless, it should be noted that the generalizability of the current study should be limited to a certain degree.

Confidence in cumulative evidence

The certainty of the results will be judged using the same classification as the GRADE (Welch et al., 2017) approach (i.e., very low, low, moderate, high). It should be noted that there is no system equivalent to GRADE in psychology that can be used to transparently rate and judge the certainty of the results. Therefore we will only utilize the classification and general ideas of GRADE.

Answer the following final questions:

Has data collection begun for this project?

- ~~No, data collection has not begun~~
- **Yes, data collection is underway or complete.**
  Specifically, the official protocol finalization started on 03/13/2020 and finished on 06/13/2020. The coding started on the same day and plans to finish by 09/11/2020.

If data collection has begun, have you looked at the data?

- ~~Yes~~
- **No**

The (estimated) start and end dates for this project are (optional):

Start date: 2020/12/11

End date: 2021/05/01

Any additional comments before I pre-register this project (optional):

No additional comments.

REFERENCES

Bartoš, F., & Schimmack, U. (2020). *Z-Curve.2.0: Estimating Replication Rates and Discovery Rates*. https://doi.org/10.31234/osf.io/urgtn

Brunner, J., & Schimmack, U. (2020). Estimating Population Mean Power Under Conditions of Heterogeneity and Selection for Significance. *Meta-Psychology*, *4*. https://doi.org/10.15626/MP.2018.874

Ikeda, K., & Hiraishi, K. (2016). The reproducibility crisis in psychology : Its structure and solutions. *Japanese Psychological Review*, *59*(1), 1–12.

Lakens, D. (2020, March 12). *What's a family in family-wise error control?* http://daniellakens.blogspot.com/2020/03/whats-family-in-family-wise-error.html

Miura, A., Okada, K., & Shimizu, H. (2018). Editorial: Make Statistics Great Again. *JAPANESE PSYCHOLOGICAL REVIEW*, *61*(1), 1–2. https://doi.org/10.24602/sjpr.61.1_1

Miura, A., Tomonaga, M., Harada, E. T., Yamada, Y., & Takezawa, M. (2019). Editorial: The new style of psychological research: CHANGE we can believe in. *Japanese Psychological Review*, *62*(3), 197–204. https://psyarxiv.com/z5cns/download?format=pdf

Open Science Collaboration. (2012). An Open, Large-Scale, Collaborative Effort to Estimate the Reproducibility of Psychological Science. *Perspectives on Psychological Science: A Journal of the Association for Psychological Science*, *7*(6), 657–660. https://doi.org/10.1177/1745691612462588

Open Science Collaboration. (2015). Estimating the reproducibility of psychological science. *Science*, *349*(6251), aac4716. https://doi.org/10.1126/science.aac4716

Schimmack, U. (2020). A meta-psychological perspective on the decade of replication failures in social psychology. *Canadian Psychology/Psychologie Canadienne*, *61*(4), 364–376. https://doi.org/10.1037/cap0000246

Simmons, J. P., Nelson, L. D., & Simonsohn, U. (2011). False-positive psychology: undisclosed flexibility in data collection and analysis allows presenting anything as significant. *Psychological Science*, *22*(11), 1359–1366. https://doi.org/10.1177/0956797611417632

Simonsohn, U., Nelson, L. D., & Simmons, J. P. (2014a). P-curve: A key to the file-drawer. *Journal of Experimental Psychology. General*, *143*(2), 534–547. https://doi.org/10.1037/a0033242

Simonsohn, U., Nelson, L. D., & Simmons, J. P. (2014b). p-Curve and Effect Size: Correcting for Publication Bias Using Only Significant Results. *Perspectives on Psychological Science: A*

Journal of the Association for Psychological Science, *9*(6), 666–681. https://doi.org/10.1177/1745691614553988

Simonsohn, U., Simmons, J. P., & Nelson, L. D. (2015). Better P-curves: Making P-curve analysis more robust to errors, fraud, and ambitious P-hacking, a Reply to Ulrich and Miller (2015). *Journal of Experimental Psychology. General*, *144*(6), 1146–1152. https://doi.org/10.1037/xge0000104

Sorić, B. (1989). Statistical "Discoveries" and Effect-Size Estimation. *Journal of the American Statistical Association*, *84*(406), 608–610. https://doi.org/10.1080/01621459.1989.10478811

Tomonaga, M., Miura, A., & Haryu, E. (2016). Editorial: Reproducibility of psychology. *JAPANESE PSYCHOLOGICAL REVIEW*, *59*(1), 1–2. https://doi.org/10.24602/sjpr.59.1_1

Ulrich, R., & Miller, J. (2015). p-hacking by post hoc selection with multiple opportunities: Detectability by skewness test?: Comment on Simonsohn, Nelson, and Simmons (2014). *Journal of Experimental Psychology. General*, *144*(6), 1137–1145. https://doi.org/10.1037/xge0000086

van Aert, R. C. M., Wicherts, J. M., & van Assen, M. A. L. M. (2016). Conducting Meta-Analyses Based on p Values: Reservations and Recommendations for Applying p-Uniform and p-Curve. *Perspectives on Psychological Science: A Journal of the Association for Psychological Science*, *11*(5), 713–729. https://doi.org/10.1177/1745691616650874

van Assen, M. A. L. M., van Aert, R. C. M., & Wicherts, J. M. (2015). Meta-analysis using effect size distributions of only statistically significant studies. *Psychological Methods*, *20*(3), 293–309. https://doi.org/10.1037/met0000025

Welch, V. A., Akl, E. A., Guyatt, G., Pottie, K., Eslava-Schmalbach, J., Ansari, M. T., de Beer, H., Briel, M., Dans, T., Dans, I., Hultcrantz, M., Jull, J., Katikireddi, S. V., Meerpohl, J., Morton, R., Mosdol, A., Petkovic, J., Schünemann, H. J., Sharaf, R. N., … Tugwell, P. (2017).

GRADE equity guidelines 1: considering health equity in GRADE guideline development: introduction and rationale. *Journal of Clinical Epidemiology*, *90*, 59–67. https://doi.org/10.1016/j.jclinepi.2017.01.014
