# Supplementary material for: A systematic review of conference papers presented at two large Japanese psychology conferences in 2013 and 2018: did Japanese social psychologists selectively report p < 0.05 results without peer review?
Source: PeerJ. 2024 Jan 18;12:e16763. doi: 10.7717/peerj.16763 (PMC10800096; doi:10.7717/peerj.16763)
Supplement: Supplemental Information 3 — We pre-registered to conduct p-curve analyses but decided not to include the results in the paper’s main text. It became clear that the p-curve analyses were redundant since the z-curve analyses could cover the research questions we originally intended to examine with the p-curve analyses. Here we report the pre-registered analysis plan and the results of the p-curve analyses. The results were consistent with the findings of the z-curve analyses. [file peerj-12-16763-s003.pdf]

Supplementary 4: P-curve analyses

We pre-registered to conduct *p*-curve analyses but decided not to include the results in the paper's main text. It became clear that the *p*-curve analyses were redundant since the *z*-curve analyses could cover the research questions we originally intended to examine with the *p*-curve analyses. Specifically, we planned to test the evidential value of a set of studies using *p*-curve analysis. We tested whether the observed *p*-value (< 0.05) distribution was significantly different from the distribution expected under the null effect. The same test could be performed with greater accuracy by examining whether the 95% confidence interval of the expected replication rate (ERR) estimate from the corresponding *z*-curve analysis does not include 5%. Likewise, another analysis plan, the 33% power test that examines whether a set of studies had at least 33% power, can be substituted with *z*-curve analysis by looking at whether the 95CI of the ERR included the 33%. Further, whereas we cannot directly compare the 2013 and the 2018 results with *p*-curve analysis, it is possible with *z*-curve analysis by comparing the ERR in 2013 with that in 2018. Here we report the analysis plan and the results of the pre-registered *p*-curve analyses. The results were consistent with the findings of z-curve analyses.

Analysis plan

Primary analyses

With *p*-curve analysis, we can test whether a set of studies has evidential value. Specifically, if a set of studies has evidential value, the *p*-curve (distribution of reported *p*-values smaller than .05) should be right-skewed. Conversely, if the effect in question is null, the *p*-curve should show a uniform distribution. Moreover, if researchers resort to *p*-hacking to acquire $p < .05$, more *p*-values will accumulate just under the .05 criterion (e.g., $p = .048$), leading to a left-skewed *p*-curve. Given this logic, the original *p*-curve paper was proposed to test the skewness of the full *p*-curve (distribution of all *p*-values under .05). However, several weaknesses of the original idea have been pointed out (Ulrich & Miller, 2015). For instance, the full *p*-curve analysis is vulnerable to “ambitious” *p*-hackers who try to have *p*-values much smaller than .05 (e.g., $p < .03$). The “Better *p*-curve” has been proposed (Simonsohn et al., 2015) and implemented on the website (*p*-curve.com) to tackle the problem by utilizing the half *p*-curve (distribution of *p*-values under .025).

We followed the recommendations of the Better *p*-curve. First, we tested the right-skewness of the full and half *p*-curves. When the half *p*-curve test is right-skewed with $p < .05$, or when both the full and half *p*-curves are right-skewed with $p < .10$, we conclude that the set of studies has evidential value. When the right-skewness tests turned out to be non-significant, we proceeded to the 33% power test to see if the *p*-curve was flatter than expected if the studies were powered at 33%. As the shape of the *p*-curve depends on the power, the p-curve of studies with 33% power would be fairly flat, albeit right-skewed. If the observed *p*-curve is significantly flatter than the 33% *p*-curve, we would conclude that the set of studies lacks evidential value. To be precise, following the description on *p*-curve.com, we would conclude that “*evidential value is inadequate or absent if the 33% power test is p < .05 for*

the full p-curve or both the half p-curve and binomial 33% power test are p < .1." Note that the binomial test examines the share of *p*-values smaller than .025 among all *p*-values under .05).

The *p*-values included in the *p*-curve analysis should be independent of each other. In addition, the proposed *p*-curve analysis strongly recommended re-calculating *p*-values from *p*-stats (e.g., *t*-values and DFs) rather than relying on *p*-values reported in the target articles (Simonsohn et al., 2015). Therefore, we used the *p*-stats first appearing in each conference paper to conduct *p*-curve analyses (Fig. S4-1). We used the *p*-curve app 4.06, available at *p*-curve.com, for the analyses (Simonsohn et al., 2017).

Figure S4-1: Examples of p-stats coding format for p-curve.com

```
t(88)=2.1
r(147)=.246
F(1,100)=9.1
f(2,210)=4.45
Z=3.45
chi2(1)=9.1
r(77)=.47
chi2(2)=8.74
```

Family-wise error control

For the *p*-curve analysis, we planned to conduct several null hypothesis significance tests. We analyzed the 2013 and 2018 data separately. We conducted several right-skewness tests for each year with different alpha levels. In addition, when these tests do not reach significance, we would proceed to the 33% power tests, including several null hypothesis tests with different alpha levels. As such, controlling for the family-wise error rate is complicated. Therefore, we decided not to declare any family-wise error corrections beforehand. Instead, we reported all tests and raw *p*-values from the *p*-curve analyses. If the findings are not robust, they should eventually appear in the results (e.g., only a small portion of the tests are significant) (Lakens, 2020).

Sensitivity analyses

Several conference papers have reported more than two *p*-stats. We used the *p*-stats that appeared last in the papers for the sensitivity analyses[1]. Thus, when there was only one eligible *p*-stats in a paper, it was used for the sensitivity analysis. If there were more, we took the last one.

[1] We pre-registered to use the second reported *p*-stats for the sensitivity analyses. However, it turned out that picking up the last *p*-stats required much simpler R-script, making the possibility of errors much smaller. Therefore, we decided to use the last *p*-stats for sensitivity analyses.

Results

Primary analyses

The criterion for evidential value was that either the half *p*-curve test was right-skewed with $p <. 05$ or both the half and full tests were right-skewed with $p < .10$. These conditions were consistent with the papers in 2013. Both the half *p*-curve and full p-curve were right-skewed with $p < .0001$ (Z = -11.31 and Z = -13.95, respectively). In addition, the 33% power test, which would indicate a lack of evidential value when the observed *p*-curve was flatter than the 33% power *p*-curve, was not significant for either the half *p*-curve or the full *p*-curve (Z = 5.55, p > .999; Z = 15.09, p > .999, respectively). Therefore, the *p*-curve analysis indicated the presence of an evidential value in 2013 (Fig. S4-2).

Likewise, for papers in 2018, we found that both the half *p*-curve and full *p*-curve were right-skewed with $p < .0001$ (Z = -13.08 and Z = -15.20, respectively). The 33% power test was not significant for either the half *p*-curve or the full *p*-curve (Z = 7.86, p > .999; Z = 14.40, p > .999, respectively). Therefore, the evidential value is also indicated in the papers in 2018 (Fig. S4-3). Notably, the *p*-curve of the 2018 study appears to be more right-skewed than that of the 2013 study. Right-skewness tests showed larger absolute Z values for the half *p*-curves (Z = -11.31 and Z = -13.08) and the full *p*-curves (Z = -13.95 and Z = -15.20) in 2018 than in 2013.

Sensitivity analyses

We conducted sensitivity analyses with the last *p*-stats reported in a paper separately for 2013 and 2018. Sensitivity analyses with the last *p*-stats reported in a paper also showed evidential value for the 2013 and 2018 papers. All relevant right-skewness tests were significant, whereas the 33% power tests were not. Therefore, it was shown that the set of studies in 2013 and 2018 had evidential value. Figures S4-4 and S4-5 are copy-and-paste of the outputs produced by p-curve app 4.06, available at p-curve.com.

Conclusion

The *p*-curve analysis showed that the set of studies in both years had evidential values. The distributions of *p*-values under 5% were significantly right-skewed (Fig. S4-2 to S4-5) and were not significantly flatter than the *p*-curve of studies with 33% power in 2013 and 2018. The conference papers presented at the two Japanese psychology societies had, as a whole, certain levels of evidential value. Notably, the *p*-curve of the 2018 studies appeared to be more right-skewed than those of the 2013 studies. This suggests that the set of studies in 2018 had a stronger evidential value than those in 2013. These were consistent with findings from the *z*-curve analyses reported in the main text.

Figure S4-2: P-curve of the papers in 2013 produced by p-curve 4.06

The observed *p*-curve includes 71 statistically significant (p < .05) results, of which 45 are *p* < .025. There were 20 additional results entered but excluded from *p*-curve because they were *p* > .05.

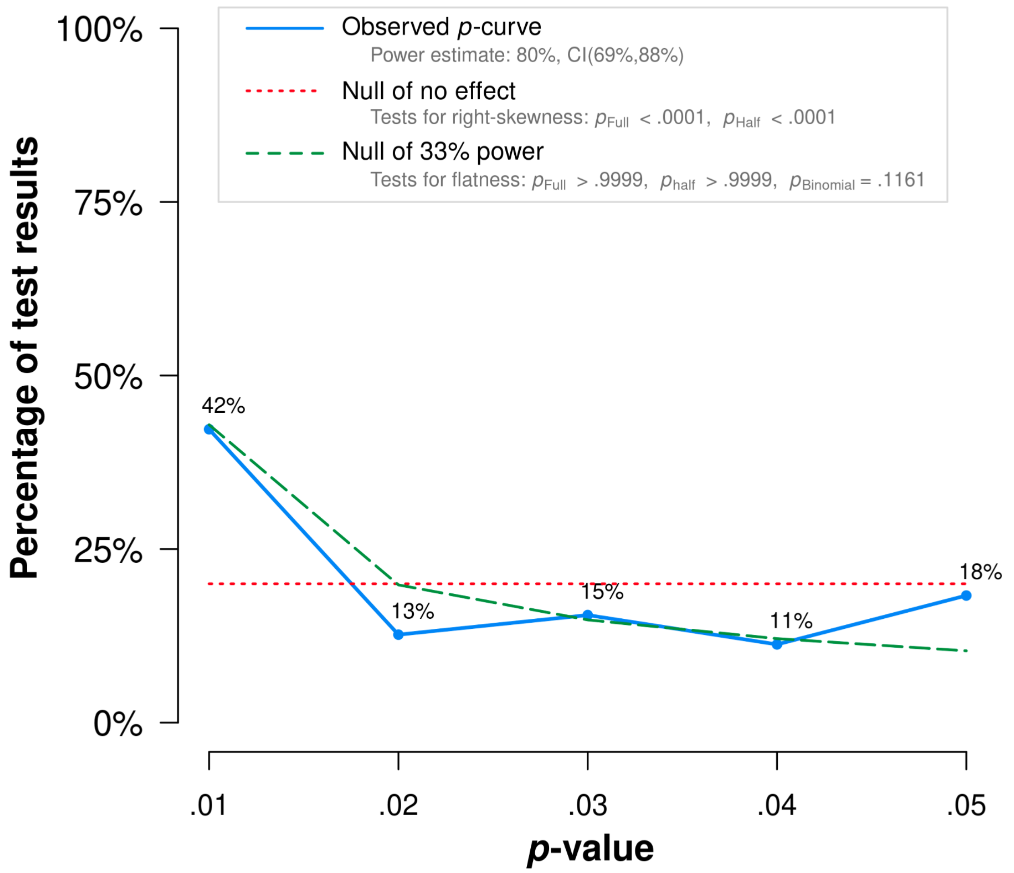

	Binomial Test<br>*(Share of results p<.025)*	Continuous Test<br>*(Aggregate with Stouffer Method)*	
		**Full p-curve (p's<.05)**	**Half p-curve (p's<.025)**
1) Studies contain evidential value. *(Right skew)*	*p*=.016	*Z*=-11.31, *p*<.0001	*Z*=-13.95, *p*<.0001
2) Studies' evidential value, if any, is inadequate. *(Flatter than 33% power)*	*p*=.1161	*Z*=5.55, *p*>.9999	*Z*=15.09, *p*>.9999
	**Statistical Power**		
Power of tests included in *p*-curve *(correcting for selective reporting)*	Estimate: 80%<br>90% Confidence interval: (69% , 88%)		

Figure S4-3: P-curve of the papers in 2018 produced by p-curve 4.06

The observed p-curve includes 42 statistically significant ($p < .05$) results, of which 32 are $p < .025$. There were 24 additional results entered but excluded from p-curve because they were $p > .05$.

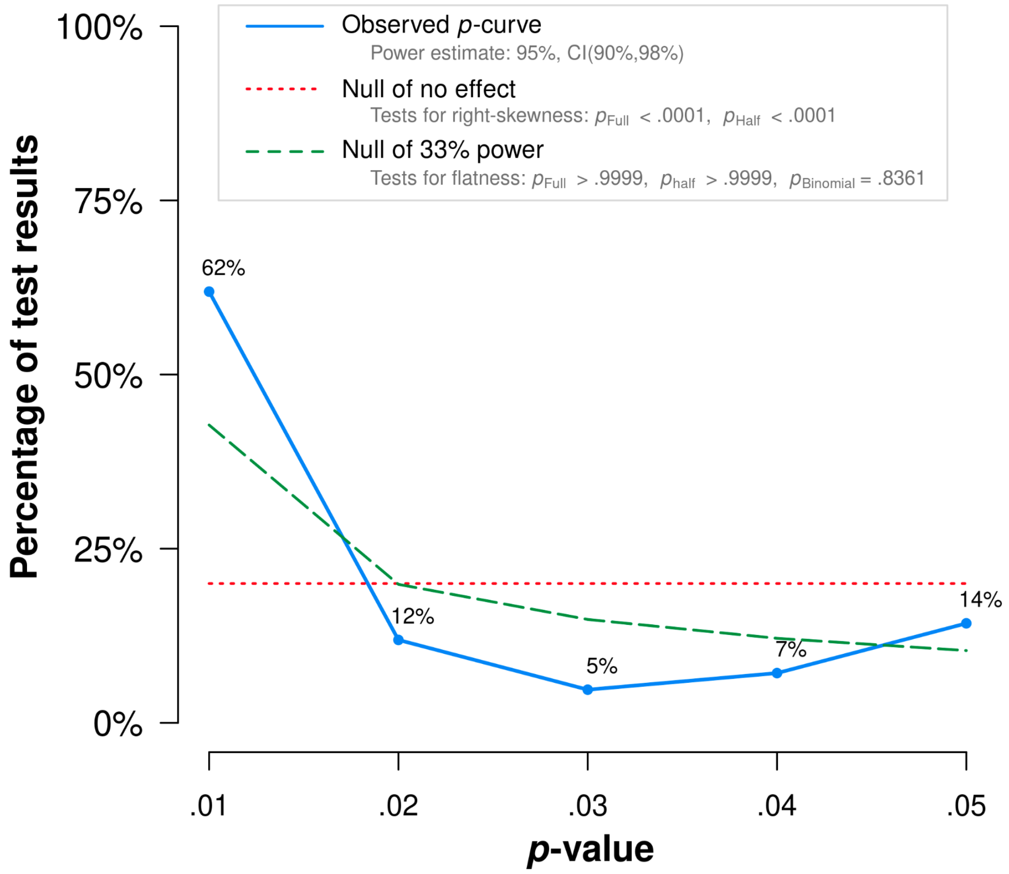

	Binomial Test (Share of results p<.025)	Continuous Test (Aggregate with Stouffer Method)	
		Full p-curve (p's<.05)	Half p-curve (p's<.025)
1) Studies contain evidential value. *(Right skew)*	p=.0005	Z=-13.08, p<.0001	Z=-15.2, p<.0001
2) Studies' evidential value, if any, is inadequate. *(Flatter than 33% power)*	p=.8361	Z=7.86, p>.9999	Z=14.4, p>.9999
	**Statistical Power**		
Power of tests included in *p*-curve *(correcting for selective reporting)*	Estimate: 95%<br>90% Confidence interval: (90% , 98%)		

Figure S4-4. P-curve analysis of 2013 data (sensitivity analysis with last p-stats)

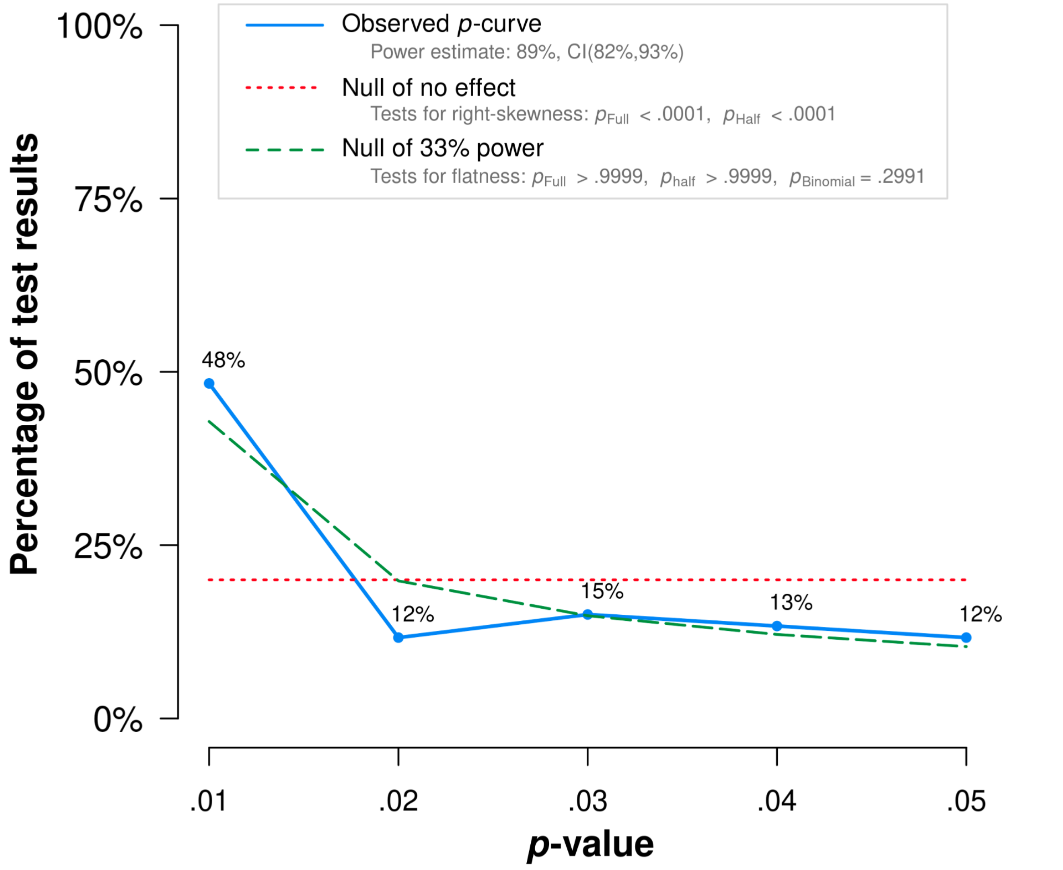

Note: The observed *p*-curve includes 60 statistically significant ($p < .05$) results, of which 40 are $p < .025$. There were 28 additional results entered but excluded from *p*-curve because they were $p > .05$.

	**Binomial Test** *(Share of results p<.025)*	**Continuous Test** *(Aggregate with Stouffer Method)*	
		**Full p-curve (p's<.05)**	**Half p-curve (p's<.025)**
1) Studies contain evidential value. *(Right skew)*	p=.0067	Z=-12.9, p<.0001	Z=-14.99, p<.0001
2) Studies' evidential value, if any, is inadequate. *(Flatter than 33% power)*	p=.2991	Z=7.42, p>.9999	Z=15.32, p>.9999
	**Statistical Power**		
Power of tests included in *p*-curve *(correcting for selective reporting)*	Estimate: 89%<br>90% Confidence interval: (82% , 93%)		

Figure S4-5. P-curve analysis of 2018 data (sensitivity analysis with last p-stats)

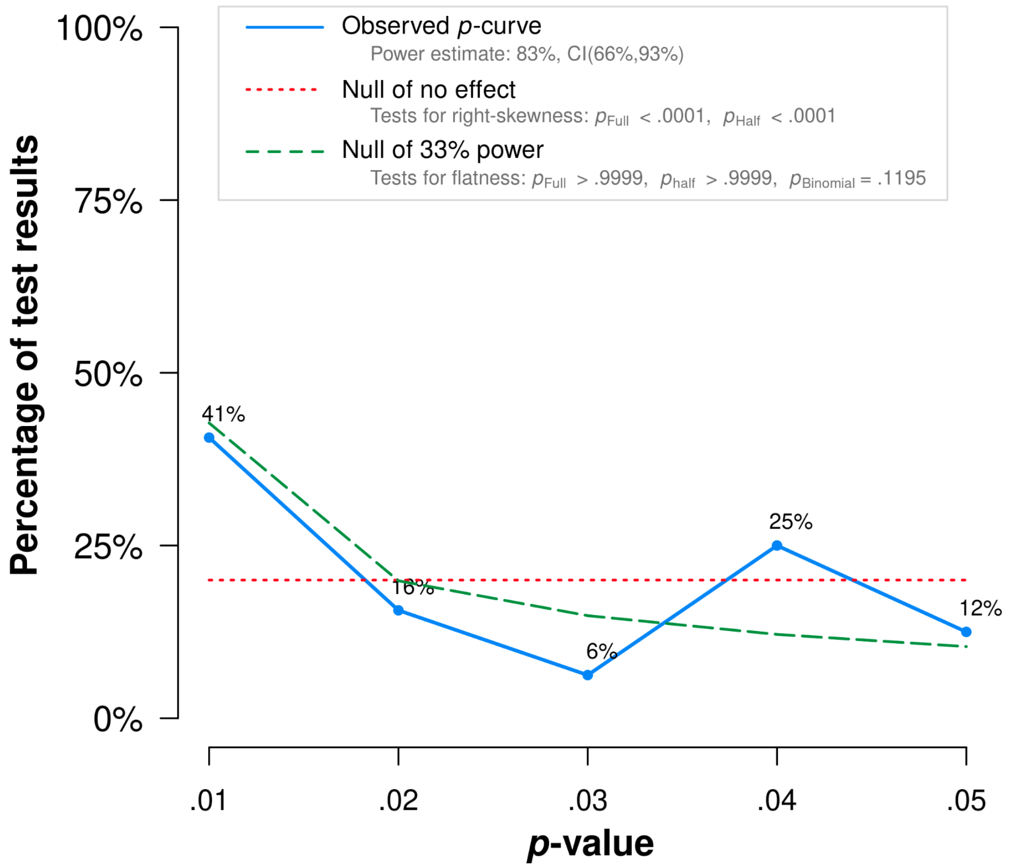

Note: The observed *p*-curve includes 32 statistically significant ($p < .05$) results, of which 19 are $p < .025$. There were 31 additional results entered but excluded from *p*-curve because they were $p > .05$.

	Binomial Test<br>*(Share of results p<.025)*	Continuous Test<br>*(Aggregate with Stouffer Method)*	
		Full p-curve<br>(p's<.05)	Half p-curve<br>(p's<.025)
1) Studies contain evidential value.<br>*(Right skew)*	*p*=.1885	*Z*=-7.89, *p*<.0001	*Z*=-10.52, *p*<.0001
2) Studies' evidential value, if any, is inadequate.<br>*(Flatter than 33% power)*	*p*=.1195	*Z*=3.74, *p*=.9999	*Z*=10.49, *p*>.9999
	**Statistical Power**		
Power of tests included in *p*-curve<br>*(correcting for selective reporting)*	Estimate: 83%<br>90% Confidence interval: (66% , 93%)		

References

Lakens, D. (2020, March 12). What's a family in family-wise error control? http://daniellakens.blogspot.com/2020/03/whats-family-in-family-wise-error.html

Simonsohn, U., Nelson, L. D., & Simmons, J. P. (2014a). P-curve: A key to the file-drawer. *Journal of Experimental Psychology. General*, *143*(2), 534–547. https://doi.org/10.1037/a0033242

Simonsohn, U., Nelson, L. D., & Simmons, J. P. (2014b). p-Curve and Effect Size: Correcting for Publication Bias Using Only Significant Results. *Perspectives on Psychological Science: A Journal of the Association for Psychological Science*, *9*(6), 666–681. https://doi.org/10.1177/1745691614553988

Simonsohn, U., Nelson, L. D., & Simmons, J. P. (2017, November 30). *The p-curve app 4.06*. P-Curve.com. http://www.p-curve.com/app4/

Simonsohn, U., Simmons, J. P., & Nelson, L. D. (2015). Better P-curves: Making P-curve analysis more robust to errors, fraud, and ambitious P-hacking, a Reply to Ulrich and Miller (2015). *Journal of Experimental Psychology. General*, *144*(6), 1146–1152. https://doi.org/10.1037/xge0000104

Ulrich, R., & Miller, J. (2015). *p*-hacking by post hoc selection with multiple opportunities: Detectability by skewness test?: Comment on Simonsohn, Nelson, and Simmons (2014). *Journal of Experimental Psychology. General*, *144*(6), 1137–1145. https://doi.org/10.1037/xge0000086
