# Supplementary material for: A systematic review of conference papers presented at two large Japanese psychology conferences in 2013 and 2018: did Japanese social psychologists selectively report p < 0.05 results without peer review?
Source: PeerJ. 2024 Jan 18;12:e16763. doi: 10.7717/peerj.16763 (PMC10800096; doi:10.7717/peerj.16763)
Supplement: Supplemental Information 4 — We conducted several sensitivity analyses with the z-curve analysis. First, there were three different methods of estimation; Expectancy-Maximization (EM), Kernel Density 1 (KD1), and Kernel Density 2 (KD2). Second, there were several sets of data. For the primary analyses, we employed either p-values or p-stats (statistical information sufficient to compute the p-value) that appeared first in a paper (first p-stats/p-value). For sensitivity analyses, we employed p-stats/p-value that appeared last in a paper (last p-stats/p-value). We also employed the first and last p-stats only as we did with the p-curve analyses. The results are summarized in the following tables (Table S5-1 to S5-4). In the following, ERR denotes the Expected Replication Rate; EDR denotes the Expected Discovery Rate; FDR denotes the maximum False Discovery Rate; ODR denotes the Observed Discovery Rate. [file peerj-12-16763-s004.pdf]

Supplementary 5

We conducted several sensitivity analyses with the *z*-curve analysis. First, there were three different methods of estimation; Expectancy-Maximization (EM), Kernel Density 1 (KD1), and Kernel Density 2 (KD2). Second, there were several sets of data. For the primary analyses, we employed either *p*-values or *p*-stats (statistical information sufficient to compute the *p*-value) that appeared first in a paper (first *p*-stats/*p*-value). For sensitivity analyses, we employed *p*-stats/*p*-value that appeared last in a paper (last *p*-stats/*p*-value). We also employed the first and last *p*-stats only as we did with the *p*-curve analyses. The results are summarized in the following tables (Table S5-1 to S5-4). In the following, ERR denotes Expected Replication Rate; EDR denotes Expected Discovery Rate; FDR denotes the maximum False Discovery Rate; ODR denotes Observed Discovery Rate.

Table S5-1. ***Z*-curve analyses with papers in 2013 either with first or last *p*-stats/*p*-values**

		p-stats/*p*-values						
	mean	First				Last		
method	power	estimate	lower	upper		estimate	lower	upper
EM	ERR	0.31	0.16	0.48		0.35	0.21	0.53
	EDR	0.07	0.05	0.15		0.10	0.05	0.32
	FDR	0.68	0.30	1.00		0.49	0.11	1.00
KD2	ERR	0.33	0.15	0.47		0.38	0.21	0.58
	EDR	0.07	0.05	0.24		0.17	0.05	0.35
	FDR	0.67	0.17	1.00		0.40	0.10	1.00
KD1	ERR	0.34	0.14	0.49		0.39	0.210	0.60
Observed	ODR	0.76	0.66	0.84		0.66	0.56	0.75

Table S5-2. ***Z*-curve analyses with papers in 2013 either with first or last *p*-stats**

		p-stats						
	mean	First				Last		
method	power	estimate	lower	upper		estimate	lower	upper
EM	ERR	0.33	0.17	0.48		0.37	0.21	0.56
	EDR	0.07	0.05	0.15		0.08	0.05	0.28
	FDR	0.67	0.30	1.00		0.59	0.13	1.00
KD2	ERR	0.34	0.15	0.50		0.39	0.20	0.59
	EDR	0.07	0.05	0.25		0.10	0.05	0.33
	FDR	0.66	0.16	1.00		0.46	0.11	1.00
KD1	ERR	0.34	0.15	0.52		0.40	0.21	0.63
Observed	ODR	0.78	0.68	0.86		0.68	0.57	0.77

Table S5-3. ***Z*-curve analyses with papers in 2018 with first and last *p*-stats/*p*-values**

		p-stats/*p*-values						
	mean	First				Last		
method	power	estimate	lower	upper		estimate	lower	upper
EM	ERR	0.44	0.22	0.62		0.21	0.05	0.39
	EDR	0.13	0.05	0.57		0.07	0.05	0.24
	FDR	0.35	0.04	1.00		0.73	0.17	1.00
KD2	ERR	0.50	0.29	0.69		0.24	0.07	0.46
	EDR	0.17	0.05	0.64		0.08	0.05	0.28
	FDR	0.25	0.03	1.00		0.63	0.13	1.00
KD1	ERR	0.50	0.28	0.71		0.25	0.07	0.48
Observed	ODR	0.64	0.52	0.74		0.56	0.44	0.67

Table S5-4. ***Z*-curve analyses with papers in 2018 with first and last *p*-stats**

Table S5-4		*p*-stats						
	mean	First				Last		
2018	power	estimate	lower	upper		estimate	lower	upper
EM	ERR	0.49	0.27	0.68		0.26	0.07	0.50
	EDR	0.16	0.05	0.63		0.07	0.05	0.27
	FDR	0.27	0.03	1.00		0.69	0.14	1.00
KD2	ERR	0.57	0.32	0.77		0.32	0.09	0.55
	EDR	0.19	0.05	0.71		0.07	0.05	0.33
	FDR	0.22	0.02	1.00		0.68	0.11	1.00
KD1	ERR	0.58	0.33	0.77		0.32	0.09	0.60
Observed	ODR	0.64	0.51	0.75		0.51	0.38	0.63
