# Supplementary material for: A systematic review of conference papers presented at two large Japanese psychology conferences in 2013 and 2018: did Japanese social psychologists selectively report p < 0.05 results without peer review?
Source: PeerJ. 2024 Jan 18;12:e16763. doi: 10.7717/peerj.16763 (PMC10800096; doi:10.7717/peerj.16763)
Supplement: Supplemental Information 7 — Explaining the rationale and contribution of the current systematic review. [file peerj-12-16763-s007.pdf]

Supplementary 8

1. The rationale for conducting the systematic review/meta-analysis

The purpose of the current systematic review and the meta-analysis was to test a hypothesis that social psychologists internalized the cultural norm of the field to prioritize the statistical significance of results and voluntarily refrained from reporting non-significant results.

It is often argued that psychologists selectively report significant results to meet journal preferences, and such behavior contributed to the replicability crisis in psychology (Bakker et al., 2012; Nosek et al., 2012). In fact, several meta-analytical studies have reported the existence of publication bias in psychology where statistically significant results were much more likely to be published (Carter & McCullough, 2014; Chen et al., 2022; Maier, Bartoš, Oh, et al., 2022; Maier, Bartoš, Stanley, et al., 2022; McAuliffe et al., 2021; Shanks et al., 2015; Sotola & Credé, 2022). However, it is also possible that authors voluntarily refrained from reporting non-significant results regardless of the journal preferences. As long as we analyze published studies, we cannot discriminate between publication bias made by the journal (editors and reviewers) and that made on the authors' side.

The conference papers at two large Japanese psychology conferences allowed us to test the possibility that authors voluntarily prioritized reporting significant results (i.e., $p < .05$ results). As authors of poster presentations at the conferences could report almost anything without fear of rejection by peer reviewers, if we find publication bias among them, it clearly shows the preference on the authors' side. To our knowledge, no meta-analysis has been conducted on studies like this. Thus, the current systematic review and meta-analysis would provide valuable evidence to ascertain whether psychologists were forced by journal pressure to selectively report significant results or whether they had such preferences themselves.

2. The contribution that it makes to knowledge in light of previously published related reports, including other meta-analyses and systematic reviews.

The current study contributes to adding valuable knowledge about publication bias on the part of authors. As mentioned above, a number of meta-analysis studies have reported the existence of publication bias among psychology papers. However, these studies could not discriminate between publication bias on the journal side and that on the authors' side. A notable exception is Franco et al., (2014), which examined the outcome of research proposals that had undergone rigorous peer review and found that even such proposals were less likely to be written up when the results were negative. The current systematic review and meta-analysis targeted a wider group

of studies: All presentations by Japanese social psychologists at major conferences in given years that reported experiments with one or more directed hypotheses. As such, the current study contributes to improving the understanding of publication bias on the part of authors by utilizing a rare and valuable resource that Japanese psychological societies have accumulated.

References

Bakker, M., van Dijk, A., & Wicherts, J. M. (2012). The Rules of the Game Called Psychological Science. *Perspectives on Psychological Science: A Journal of the Association for Psychological Science*, *7*(6), 543–554. https://doi.org/10.1177/1745691612459060

Carter, E. C., & McCullough, M. E. (2014). Publication bias and the limited strength model of self-control: has the evidence for ego depletion been overestimated? *Frontiers in Psychology*, *5*, 823. https://doi.org/10.3389/fpsyg.2014.00823

Chen, L., Benjamin, R., Lai, A., & Heine, S. (2022). *Managing the terror of publication bias: A comprehensive p-curve analysis of the Terror Management Theory literature*. https://doi.org/10.31234/osf.io/kuhy6

Franco, A., Malhotra, N., & Simonovits, G. (2014). Social science. Publication bias in the social sciences: unlocking the file drawer. *Science*, *345*(6203), 1502–1505. https://doi.org/10.1126/science.1255484

Maier, M., Bartoš, F., Oh, M., Wagenmakers, E.-J., Shanks, D., & Harris, A. J. L. (2022). *Adjusting for Publication Bias Reveals That Evidence for and Size of Construal Level Theory Effects is Substantially Overestimated*. https://doi.org/10.31234/osf.io/r8nyu

Maier, M., Bartoš, F., Stanley, T. D., Shanks, D. R., Harris, A. J. L., & Wagenmakers, E.-J. (2022). No evidence for nudging after adjusting for publication bias. *Proceedings of the National Academy of Sciences*, *119*(31), e2200300119. https://doi.org/10.1073/pnas.2200300119

McAuliffe, W. H. B., Edson, T. C., Louderback, E. R., LaRaja, A., & LaPlante, D. A. (2021). Responsible product design to mitigate excessive gambling: A scoping review and z-curve analysis of replicability. *PloS One*, *16*(4), e0249926. https://doi.org/10.1371/journal.pone.0249926

Nosek, B. A., Spies, J. R., & Motyl, M. (2012). Scientific Utopia : II. Restructuring Incentives and

Practices to Promote Truth Over Publishability. *Perspectives on Psychological Science: A Journal of the Association for Psychological Science*, *7*(6), 615–631. https://doi.org/10.1177/1745691612459058

Shanks, D. R., Vadillo, M. A., Riedel, B., Clymo, A., Govind, S., Hickin, N., Tamman, A. J. F., & Puhlmann, L. M. C. (2015). Romance, risk, and replication: Can consumer choices and risk-taking be primed by mating motives? *Journal of Experimental Psychology. General*, *144*(6), e142–e158. https://doi.org/10.1037/xge0000116

Sotola, L. K., & Credé, M. (2022). On the predicted replicability of two decades of experimental research on system justification: A Z‐curve analysis. *European Journal of Social Psychology*. https://doi.org/10.1002/ejsp.2858
